# Effect of Laser Parameters on Through-Thickness Local Hardness of Polypropylene Plates

**DOI:** 10.3390/ma18112638

**Published:** 2025-06-04

**Authors:** André Guimarães, Daniel Gomes, André Vieira, Serafim M. Oliveira

**Affiliations:** 1 CISeD—Research Centre for Digital Services, Polytechnic Institute of Viseu, Av. Cor. José Maria Vale de Andrade, 3504-510 Viseu, Portugal or andre.m.guimaraes@ubi.pt (A.G.); soliveira@estgv.ipv.pt (S.M.O.); 2 CISE—Electomechatronic Systems Research Centre, University of Beira Interior, Rua Marquês de Ávila e Bolama, 6201-001 Covilhã, Portugal; 3 Department of Mechanical Engineering and Industrial Management, Polytechnic Institute of Viseu, Av. Cor. José Maria Vale de Andrade, 3504-510 Viseu, Portugal; dani.o.gomes1@gmail.com; 4 CMAST—Centre for Mechanical and Aerospace Science and Technologies, University of Beira Interior, Rua Marquês de Ávila e Bolama, 6201-001 Covilhã, Portugal

**Keywords:** polypropylene, laser processing, polymer welding, bead characteristics

## Abstract

Laser technology is widely regarded as a highly effective method for welding thermoplastic polymers due to its precision, cleanliness, and versatility. This study investigates the effects of laser power and scanning speed on the through-thickness hardness of polypropylene plates, analyzing the heat-affected zone (HAZ) and hardness variations along the depth of the weld bead. Using the Trumpf Trudisk 6602 laser source, multiple polypropylene passes were made with different power levels (200 W and 300 W) and scanning speeds (5, 10, 20, 30, 40, and 50 mm/s). The results indicate a direct correlation between laser power and scanning speed in the final width and depth of the weld bead. Furthermore, results indicate that higher scanning speeds and lower power promote a more uniform distribution of hardness across the thickness. This study contributes to understanding laser-assisted welding processes in polymeric materials, providing information on the influence of different laser parameters on weld quality and resulting material properties.

## 1. Introduction

The necessity to join various materials is increasing as scientific research into creating new polymeric goods progresses [1]. The literature claims that because of its effectiveness, adaptability, and little pollution emissions throughout the operation, laser technology is the best option for welding thermoplastic polymers [2,3,4,5,6].

Among the available joining methods for thermoplastic polymers, such as ultrasonic welding, hot plate welding, and resistance welding, laser welding has gained particular attention due to its high precision, localized heat input, and excellent reproducibility. Unlike hot plate or resistance welding, which often requires direct contact and generates extensive heat-affected zones (HAZ), laser welding enables non-contact processing with better thermal profile control, reducing the risk of material degradation and improving weld aesthetics. Compared to ultrasonic welding, laser-based methods offer greater flexibility for complex geometries and multi-layer assemblies, and they are more compatible with automation systems, particularly in high-throughput industrial environments. Furthermore, laser welding can produce narrower weld seams and finer microstructural transitions, which are advantageous for mechanical performance. These benefits are consistent with findings from metallic systems, where laser processing has demonstrated superior structural integrity and mechanical properties, as reported by Zhang [7]. When the CO_2_ laser welding system was first launched in the early 1970s, its capacity to perform welds, particularly in polymeric materials, was emphasized [8,9].

Polymer fusion, followed by solidification, can result in various morphological changes. Semicrystalline (SC) thermoplastics, like polypropylene (PP), have both crystalline and amorphous regions [10,11]. When the laser beam strikes the polymer, it forms a continuous heat wave that causes recrystallization. Due to increased crystallinity and spherulite production, these changes in the initial polymer morphology have a direct effect on recrystallization following laser beam incidence, which lowers laser energy transmission in the polymer [12,13,14,15]. Actually, the material’s crystallinity characteristics have a significant impact on the laser welding process’s performance since they directly determine how much laser energy the polymer absorbs [16]. This phenomenon is particularly noticeable in thermoplastics, as they can be highly crystalline polymers or contain materials that disperse laser energy more widely, negatively impacting the laser transmission welding (LTW) process [17].

The thermally affected region expands and contracts as a result of the material’s local heating during the LTW process, which raises the temperature significantly. The hardness varies across the depth of the observed weld bead pools as a result of this thermal expansion and contraction. As stated in Refs. [18,19], residual stresses resulting from unrelieved thermal stresses after cooling might result in cracks and deformations.

In laser transmission welding, if the heat input is too high, the joint may be degraded due to excessive heat, which can result in reduced joint strength. On the other hand, if the heat input is too low, the joint may not be completely fused, also leading to low joint strength [17]. A larger weld zone volume and a deeper penetration are achieved by increasing the laser power and decreasing the laser beam’s scanning speed. The heat energy from the laser gets trapped in the polymers and can raise the temperature in deeper regions [20]. Higher laser power values and lower scanning speeds can degrade the morphological and optical properties of polymers [21]. In addition to laser power, scanning speed is a crucial factor for productivity in laser welding. Lower speeds result in longer exposure times to the beam, causing overheating and thermal degradation of the material, leading to weaker weld beads [13,22,23,24,25,26,27,28,29,30,31]. However, increasing laser speed results in incomplete fusion due to the low heat incident on the workpiece [32], resulting from the short irradiation time [33], leading to lower joint quality [25]. Achieving the proper power balance is crucial for laser welding. While insufficient heat results in partial fusion and decreased weld strength, excessive heat deteriorates the material and weakens the heat-affected zone (HAZ) [34,35,36]. Acherjee et al. [25] identify critical parameters to achieve good weld bead quality: laser beam power, contributing to 58% of influence; focal point distance, responsible for 31% of weld bead quality; and welding speed, with 11% impact.

Unlike other methods, the maximum laser power in CO_2_ laser welding is determined by the thickness of the material because higher power causes the weld bead to be wider and deeper [28,29,37,38]. Casalino and Ghorbel [22] conducted a study on the penetration depth of three types of materials: PP, high-density polyethylene (HDPE), and low-density polyethylene (LDPE). The research concluded that the penetration depth of the polymeric material increases when increasing laser power. Then, it is observed that the depth achieved by the laser directly affects the weld strength. It should be mentioned that a high carbon concentration occasionally prevents adequate penetration, necessitating a higher laser power, which can cause polymer degradation [39]. The quality of the weld is significantly influenced by the laser power, the scanning speed, and the diameter of the incident beam, as these parameters dictate the amount of energy absorbed [14,40]. From previous observations, it can be verified that maintaining a balance between the laser welding parameters ensures a high-quality weld joint. The interplay of laser energy on the polymer and beam scanning speed directly impacts the width of the resulting weld bead. Higher laser power increases material fusion, creating a larger melt pool. The speed of the laser beam over the polymer affects the absorbed energy, decreasing viscosity and allowing for greater flow of melted material when the speed is reduced. The fluid generated at high temperatures on the material plays a crucial role in determining the width of the melt pool, acting as a heat conductor medium to the pool walls [17]. In previous work of Kumar [41], the depth and width of the weld bead were predicted through the finite element method (FEM) used to solve the three-dimensional transient heat diffusion to simulate the thermal field equation using COMSOL multiphysics software [42,43,44]. In this work, the response surface methodology (RSM) was used to optimize the combination of laser welding parameters (laser power, scanning speed, and spot diameter) to prevent polypropylene thermal decomposition, since laser energy increases the weld strength until the critical temperature of decomposition is reached. Different wavelengths and beam shapes directly impact the quality of the weld bead, influencing the distribution of the polymer and the absorption of laser energy [17]. Therefore, it is essential to comprehend the limitations of laser welding in polymers, taking into account a number of process parameters and assessing the hardness in HAZ in addition to the weld bead widths and depths.

Understanding the influence of laser parameters on the through-thickness hardness profile is crucial for optimizing processing conditions and ensuring joint quality in polymer laser welding applications. Numerical simulation is an important tool for studying LTW. It can significantly reduce experimental time and research costs and enable the acquisition of ideal process parameters. Furthermore, it can also help to explain the welding mechanism and understand the morphological changes that take place during the process. An understanding of the material’s thermal history can be beneficial in establishing laser processing conditions that can enhance the weld quality. The work of Hu [17] reviews the numerical simulation methods applied to the LTW process to predict the temperature field, stress field, melt flow field, and thermal decomposition. However, there is still a lack of transient material models that relate these fields with recrystallization and chemical damage of semicrystalline polymers. The work of Dave [14] focuses on various parameters and phenomena such as interdiffusion and microstructural changes that occur due to the laser interaction with SC polymers (specifically PP) and presents an overview of the essential characterization techniques that help to determine the weld quality. The local mechanical properties, such as microhardness, are directly related to the local thermal history, which determines the kinetic phenomena of local chemical thermal degradation and recrystallization. This study contributes to that goal by evaluating the effects of different laser powers and scanning speeds on polypropylene samples, offering new data into the relationship between processing conditions, weld geometry, and local hardness distribution. In order to do this, polypropylene (PP), which is nontoxic, lightweight, and ideal for low-temperature applications, was adopted in this investigation. Controlled tests were carried out using different combinations of the laser parameters to identify variations in the HAZ. Lastly, a hardness analysis was performed to assess its variation along the depth of the weld bead. To the best of the author’s knowledge, this study was the first to quantify the change in microhardness over the welded plate’s thickness.

## 2. Materials and Methods

**Figure 1:**
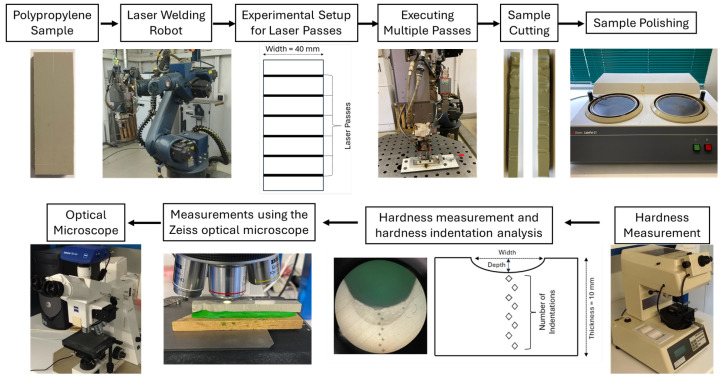
Experimental Flowchart.

The following flowchart (Figure 1) describes the experimental laser welding process on polypropylene (PP) samples used in this research. The procedure begins by selecting the supplier’s sample, which was subjected to multiple passes of a laser beam, assisted by a robot, with varying powers and speeds. After passing the laser, the sample was cut and polished to facilitate visualization of the affected areas. A microhardness tester was used to measure the hardness at different zones. An optical microscope was used to analyze the indentations and measure the width and depth of the weld beads formed.

### 2.1. Materials and Specimens

Polypropylene can be considered a semicrystalline polymer because it contains amorphous and crystalline regions. In amorphous regions, polymer chains lack order, while crystalline regions show a high level of molecular organization. Their thermal history strongly influences the degree of crystallinity and the size and type of their microstructure. The polypropylene plates used in the present work were supplied directly by the manufacturer, and their properties are listed in Table 1.

### 2.2. Laser Assisted Welding Setup

**Figure 2:**
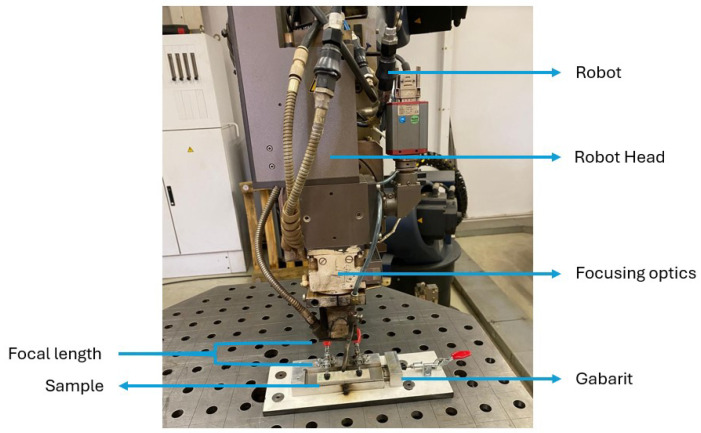
Laser welding robot making multiple passes on PP.

The laser source used in this study was the Trumpf Trudisk 6602 (TRUMPF, Schramberg, Germany) (Figure 2), generated by an Nd:YAG disk laser (TRUMPF, Schramberg, Germany) capable of reaching a maximum power of 6600 W. The laser beam was transported through an LLK fiber optic cable with a central diameter of 600 µm. A KUKA robot (TRUMPF, Schramberg, Germany) assists it with a minimum focal distance to the material’s surface (BEO D70). This model is configured with a focal distance of 10 mm and a collimation distance of 150 mm, resulting in a minimum beam diameter of 0.4 mm. Table 2 summarizes the parameters of the laser welding robot.

**Figure 3:**
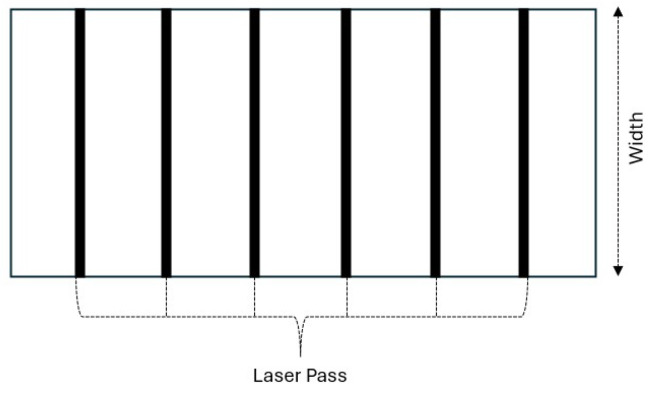
Experimental Setup for Laser Passes on Polypropylene Sample.

Figure 3 illustrates the experimental setup for laser processing of polypropylene samples. A schematic representation of the laser passes is provided, indicating multiple linear laser paths across the 40 mm width plate. This arrangement was used to study the effect of varying laser parameters, namely laser power (200 and 300 W) and scanning speeds (5, 10, 20, 30, 40, and 50 mm/s), on the hardness pattern along the plate thickness. The same focal distance of 0 mm was used in all laser passes.

### 2.3. Hardness Testing

**Figure 4:**
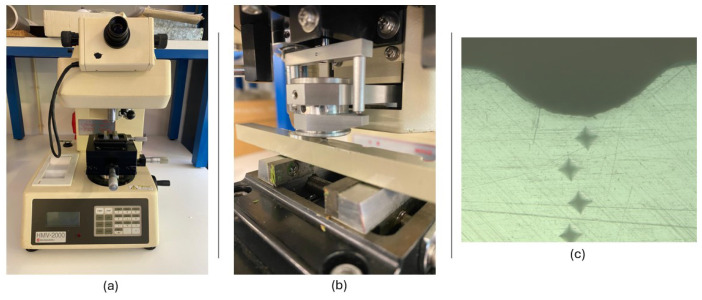
Vickers microhardness testing setup and procedure. (**a**) Vickers microhardness tester (HMV-2000, Shimadzu Corporation, Tokyo, Japan); (**b**) Diamond indenter penetrating the polypropylene surface; (**c**) Indentation marks formed on the surface of the polypropylene sample.

The hardness analysis was carried out on the entire thickness of the specimen, along the depth of the bead, using the Vickers microhardness tester (model HMV-2000 from Shimadzu Corporation, Tokyo, Japan) following the ASTM E384 standard [45] (see Figure 4a). A constant load of 0.200 kgf (1.961 N) was applied for a dwell time of 15 s using a pyramid-shaped diamond indenter (HMV-2000, Shimadzu Corporation, Tokyo, Japan) (see Figure 4b), creating an indentation mark on the material’s surface, as shown in the image of Figure 4c.

**Figure 5:**
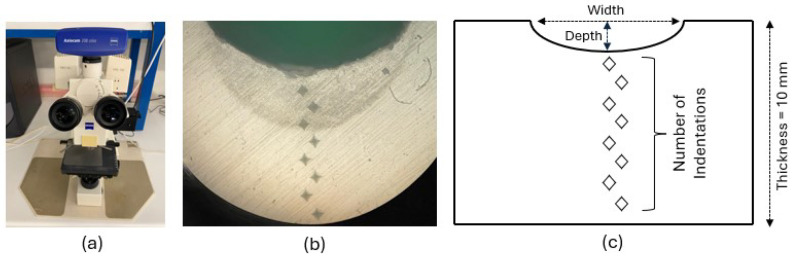
Equipment and setup for hardness measurements in laser-treated polypropylene samples. (**a**) Optical microscope used for cross-sectional analysis; (**b**) Micrograph showing Vickers indentations along the weld depth; (**c**) Schematic of indentation positions across the 10 mm thickness, indicating bead width and depth.

Due to equipment limitations, specifically the restricted field of view of the microhardness tester’s optical system, it was not possible to measure all indentations directly. Therefore, additional measurements were carried out using an optical microscope (Axiotech 100HD, Zeiss, Oberkochen, Germany) equipped with a 5× objective and imaged with a microscope extension camera (Axiocam 208 color, Carl Zeiss AG, Oberkochen, Germany) with a 0.5× lens (Figure 5a), in order to accurately capture the indentation marks that could not be measured directly with the microhardness tester (HMV-2000, Shimadzu Corporation, Tokyo, Japan). This setup enabled a detailed analysis of the cross-section of the laser-treated polypropylene plates. It allowed for accurate measurements, using a 2.5× magnification, of the weld bead’s width and depth.

Figure 5b shows a microscopic image of a welded sample, where a series of Vickers indentations can be seen aligned vertically across the thickness of the material. These indentations were used to determine the variation in hardness along the depth of the heat-affected zone (HAZ) and the fusion region.

The schematic diagram in Figure 5c illustrates the hardness testing methodology. It indicates the number and distribution of indentation points across the 10 mm thickness of the sample, as well as the geometrical parameters of the weld bead, such as width and depth. This visual representation highlights how the samples were sectioned and tested to evaluate the through-thickness hardness profile generated by different laser processing parameters.

### 2.4. Data Processing

The raw hardness values obtained from the Vickers microhardness tests were initially measured along the through-thickness direction of the polypropylene samples. Three hardness measurements were performed for each distance, starting from the upper surface. Subsequently, these values were processed to calculate the average Vickers hardness (HVavg) and the corresponding standard deviation (HVstd) for each distance from the upper surface, providing a quantitative assessment of local variability in hardness. These measured values were then analyzed and are presented in the following section.

## 3. Results and Discussion

The raw results of hardness (average and standard deviation) along the through-thickness, starting from the upper surface, are systematically presented in Table 3 and Table 4, corresponding to the tests conducted at 200 W and 300 W, respectively. The analysis focused on extracting meaningful statistical indicators such as the average Vickers hardness (HVavg) and the corresponding standard deviation (HVstd) to enable a more precise comparison between samples and experimental conditions.

**Figure 6:**
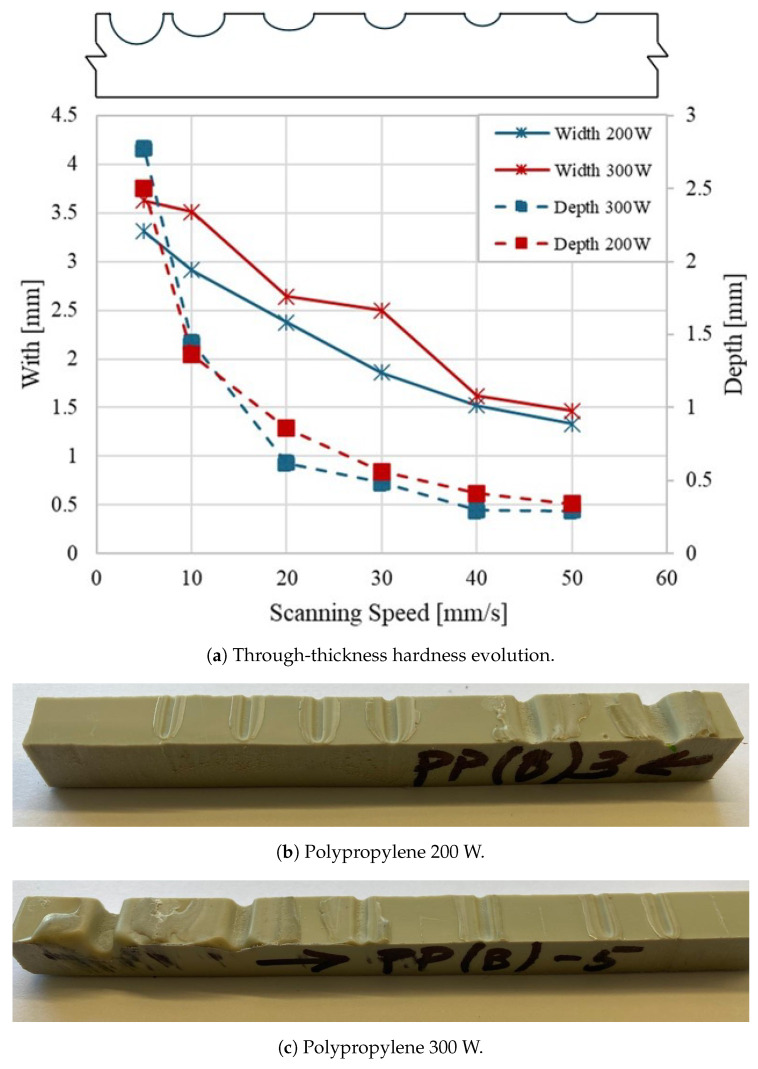
Effect of scanning speed on bead characteristics (width and depth) for polypropylene samples processed with laser treatment. (**a**) Comparison of bead width and depth at different scanning speeds; (**b**) Bead characteristics for samples processed at 200 W; (**c**) Bead characteristics for samples processed at 300 W.

Figure 6 illustrates how laser power and scanning speed affect bead characteristics in polypropylene samples. In Figure 6a, it can be seen that the bead becomes narrower and shallower as the scanning speed increases. This indicates that higher speeds lead to a more controlled and less intense thermal effect on the material. In particular, at higher scanning speeds, the differences in bead profiles for 200 W and 300 W power settings become less significant, resulting in similar bead shapes regardless of the laser power used. This behavior can also be seen in Figure 6b,c. The sample treated with 300 W shows a more pronounced bead, suggesting a more substantial thermal effect due to the higher power. In contrast, the 200 W treatment produces a more uniform bead, indicating a milder thermal impact. In general, Figure 6 highlights that adjusting the scanning speed is essential to control the thermal effect of the laser on polypropylene. Increasing the speed makes it possible to fine-tune bead width and depth, helping to achieve the desired material characteristics during laser processing.

**Figure 7:**
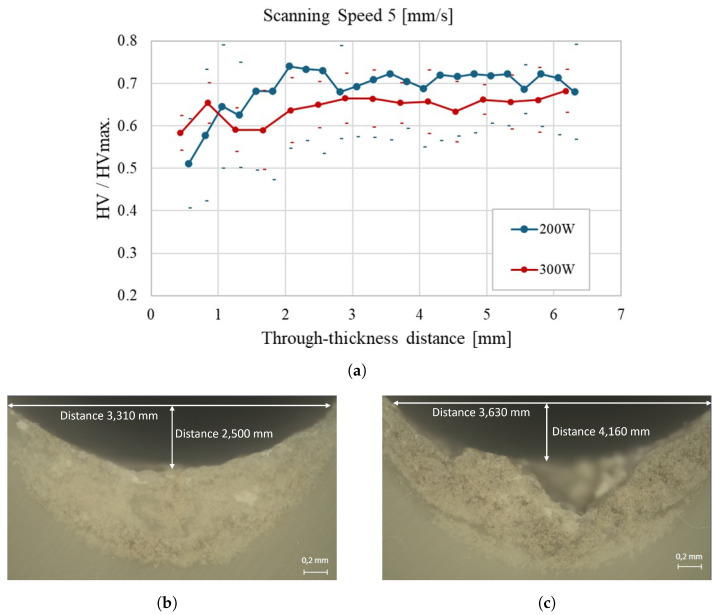
Through-thickness hardness evolution of polypropylene samples at a scanning speed of 5 mm/s: (**a**) Average hardness profiles for 200 W and 300 W laser power across the sample thickness; (**b**) Cross-sectional micrograph of the weld bead obtained at 200 W, showing a width of 3.310 mm and a depth of 2.500 mm; (**c**) Cross-sectional micrograph of the weld bead at 300 W, with a width of 3.630 mm and a depth of 4.160 mm.

Figure 7 provides a comprehensive view of how laser power influences the through-thickness hardness of polypropylene at a scanning speed of 5 mm/s. The central line represents the average values along the thickness, starting from the upper surface, while dots represent the confidence interval for each distance, considering the standard deviation of measurements. Results are presented as a fraction of the measured hardness on PP-supplied plates (14.7 V). According to the results shown in Figure 7a, using a lower laser power (200 W) results in a more uniform hardness distribution across the sample thickness compared to 300 W, which leads to more pronounced thermal alterations. This trend is supported by the micrographs: Figure 7b (200 W) shows a weld bead having a width of 3.310 mm and a depth of 2.500 mm, while Figure 7c (300 W) presents a broader and deeper bead (3.630 mm width, 4.160 mm depth), indicating a more substantial thermal effect. These findings reinforce the importance of carefully controlling laser power to optimize the hardness profile and avoid excessive thermal degradation in laser-treated polypropylene.

**Figure 8:**
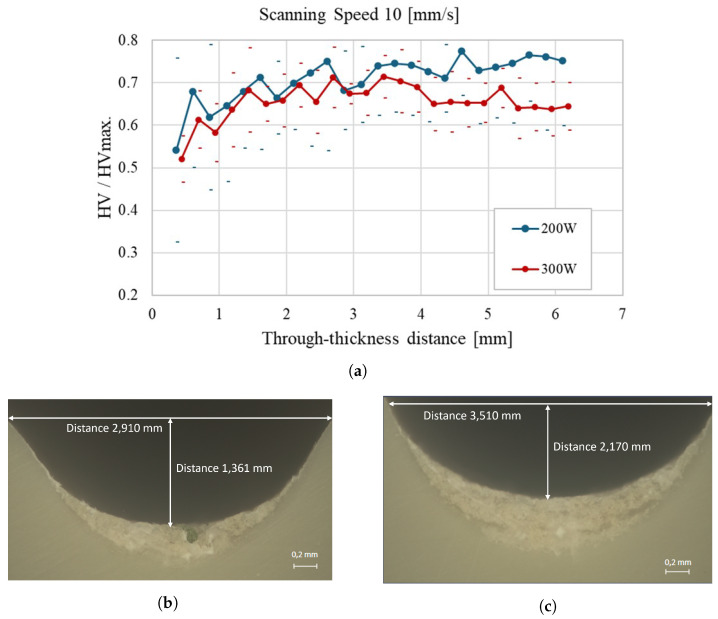
Through-thickness hardness evolution of polypropylene samples at a scanning speed of 10 mm/s: (**a**) Average hardness profiles for 200 W and 300 W laser power; (**b**) Cross-sectional micrograph of the weld bead at 200 W, with a width of 2.910 mm and a depth of 1.361 mm; (**c**) Cross-sectional micrograph of the weld bead at 300 W, with a width of 3.510 mm and a depth of 2.170 mm.

Figure 8a illustrates the impact of increasing the scanning speed to 10 mm/s on the through-thickness hardness of polypropylene. Compared to the results in Figure 7a, laser powers (200 W and 300 W) now lead to a more uniform hardness profile and slightly higher hardness values overall. The micrographs provide further insight into this behavior: In Figure 8b (200 W), the weld bead presents a width of 2.910 mm and a depth of 1.361 mm, while in Figure 8c (300 W), the bead is slightly broader and more profound, measuring 3.510 mm in width and 2.170 mm in depth. These results indicate that higher scanning speeds contribute to a more controlled thermal effect, especially at lower power, promoting a more consistent hardness distribution across the material thickness. This analysis highlights the importance of controlling scanning speed and power in laser processing to optimize material properties.

**Figure 9:**
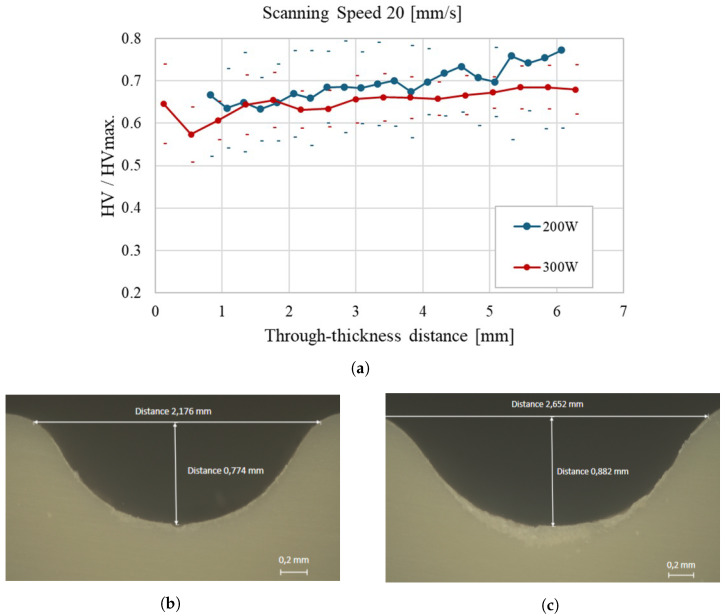
Through-thickness hardness evolution of polypropylene samples at a scanning speed of 20 mm/s: (**a**) Average hardness profiles for 200 W and 300 W laser power; (**b**) Cross-sectional micrograph of the weld bead at 200 W, with a width of 2.176 mm and a depth of 0.774 mm; (**c**) Cross-sectional micrograph of the weld bead at 300 W, with a width of 2.652 mm and a depth of 0.882 mm.

Figure 9a demonstrates that increasing the scanning speed to 20 mm/s results in a more uniform through-thickness hardness profile, particularly at 200 W. The higher scanning speed helps to minimize localized heating, promoting a smoother and more consistent increase in hardness across the sample thickness. The corresponding micrographs further confirm this behavior: in Figure 9b (200 W), the weld bead exhibits a width of 2.176 mm and a depth of 0.774 mm, while in Figure 9c (300 W), the bead is slightly larger, with a width of 2.652 mm and a depth of 0.882 mm. Although the HAZ becomes more regular at higher speeds, the results indicate that at 300 W, the laser still introduces more heat than can be evenly dissipated, leading to a slightly less uniform hardness profile than the lower power condition.

**Figure 10:**
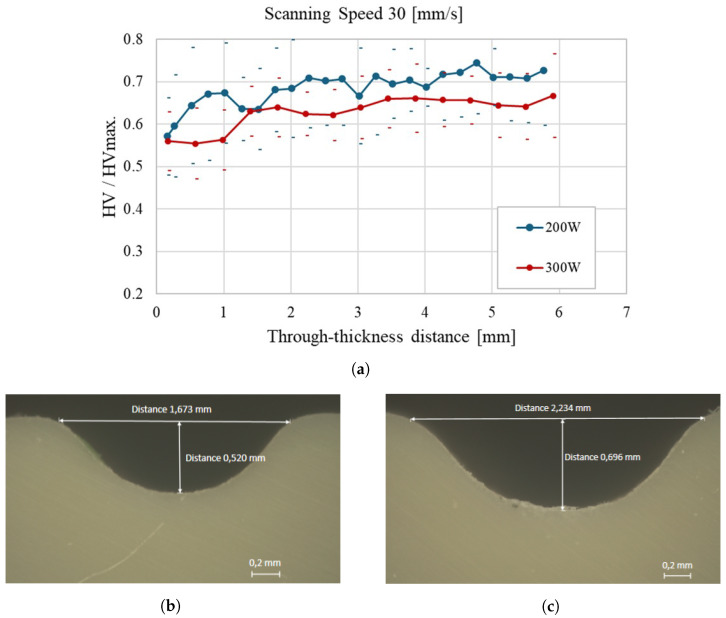
Through-thickness hardness evolution of polypropylene samples at a scanning speed of 30 mm/s: (**a**) Average hardness profiles for 200 W and 300 W laser power; (**b**) Cross-sectional micrograph of the weld bead at 200 W, with a width of 1.673 mm and a depth of 0.520 mm; (**c**) Cross-sectional micrograph of the weld bead at 300 W, with a width of 2.234 mm and a depth of 0.696 mm.

Figure 10a shows a more uniform and consistent hardness profile across the thickness of the polypropylene sample at a scanning speed of 30 mm/s, particularly under 200 W. The higher scanning speed facilitates a more gradual thermal effect, minimizing localized heating and resulting in a more homogeneous hardness distribution. This behavior is confirmed by the micrographs: in Figure 10b (200 W), the weld bead exhibits a width of 1.673 mm and a depth of 0.520 mm. In contrast, Figure 10c (300 W) shows a broader heat-affected zone, with a bead width of 2.234 mm and a depth of 0.696 mm. Although the 300 W profile still presents a gradual increase in hardness with depth, it remains slightly lower and less uniform, suggesting that excessive power leads to a more uneven thermal distribution, even at higher scanning speeds.

**Figure 11:**
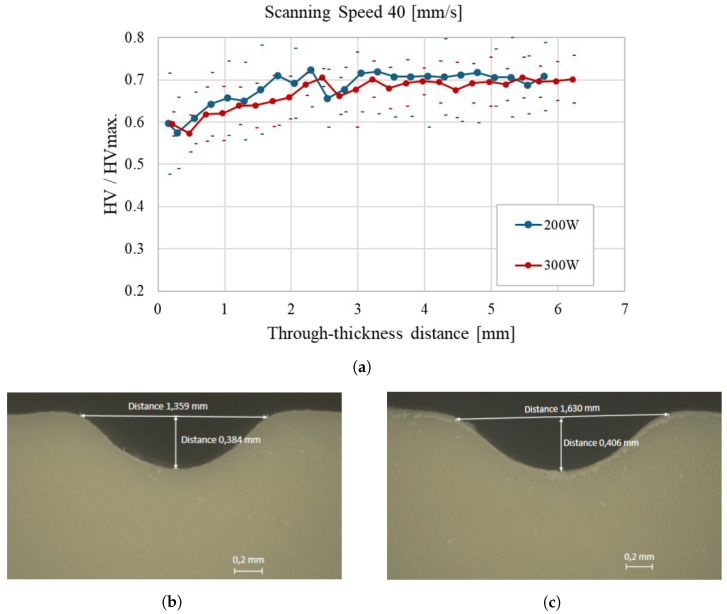
Through-thickness hardness evolution of polypropylene samples at a scanning speed of 40 mm/s: (**a**) Average hardness profiles for 200 W and 300 W laser power; (**b**) Cross-sectional micrograph of the weld bead at 200 W, with a width of 1.359 mm and a depth of 0.384 mm; (**c**) Cross-sectional micrograph of the weld bead at 300 W, with a width of 1.630 mm and a depth of 0.406 mm.

Figure 11a highlights the effect of a high scanning speed (40 mm/s) on the through-thickness hardness distribution of polypropylene samples. At 200 W, the hardness increases steadily and stabilizes across the thickness, indicating a more uniform thermal effect. In Figure 11b, the corresponding weld bead presents a width of 1.359 mm and a depth of 0.384 mm, with a narrow and well-defined heat-affected zone (HAZ). On the other hand, Figure 11c (300 W) shows a slightly larger bead (1.630 mm width, 0.406 mm depth) and a broader HAZ, reflecting a less uniform hardness profile despite the higher scanning speed. These results suggest that increasing the scanning speed helps to mitigate excessive thermal input and promotes better hardness uniformity, especially when using lower laser power.

**Figure 12:**
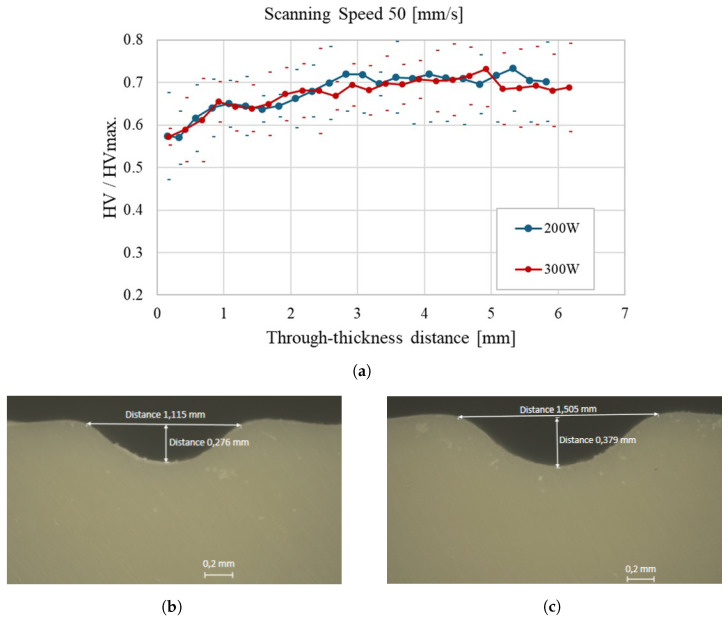
Through-thickness hardness evolution of polypropylene samples at a scanning speed of 50 mm/s: (**a**) Average hardness profiles for 200 W and 300 W laser power; (**b**) Cross-sectional micrograph of the weld bead at 200 W, with a width of 1.115 mm and a depth of 0.276 mm; (**c**) Cross-sectional micrograph of the weld bead at 300 W, with a width of 1.505 mm and a depth of 0.379 mm.

Lastly, Figure 12a shows that increasing the scanning speed to 50 mm/s further enhances the uniformity of the through-thickness hardness profile, particularly for the 200 W condition. The higher speed ensures a more controlled thermal effect, reducing localized heating and resulting in a stable and homogeneous hardness distribution across the sample thickness. The micrograph in Figure 12b (200 W) reveals a narrow weld bead, with a width of 1.115 mm and a depth of 0.276 mm, along with a well-defined and smooth heat-affected zone. In contrast, Figure 12c (300 W) displays a slightly larger bead (1.505 mm width, 0.379 mm depth), maintaining a consistent hardness profile, though slightly less uniform than at 200 W. These results confirm that higher scanning speeds achieve uniform hardness in polypropylene laser treatments, even when different power levels are applied.

A globaldecrease in hardness after the laser treatment can be attributed to chemical thermal degradation and recrystallization. For the different scanning speeds, it can be observed that the hardness curve for the higher laser power (300 W) is almost always below the hardness curve for the lower laser power (200 W). This is in accordance with the literature, which indicates that higher laser power causes greater degradation of the polymer. Unlike the strengthening effects of laser-induced structural modification observed in metallic alloys, as reported by Ouyang et al. [46], in SC polymers, the underlying mechanisms are different. In the case of SC polymers, one can obtain a reduction of strength and hardness. It is well known that polymer strength and hardness are related to the molecular weight. It can be concluded that the laser beam promotes chain scissions on the polymeric macromolecules that result in the reduction of the number average molecular weight. Furthermore, the difference between the hardness curves for 200 W and 300 W becomes less significant for higher scanning speeds. On the other hand, when exposed to laser radiation, the polymer absorbs thermal energy, particularly in the HAZ, leading to localized melting followed by solidification. This thermal cycle alters the semicrystalline microstructure of PP by promoting the reorganization of crystalline regions. As supported by the literature [12,13,17,46], these morphological transformations result in a higher crystalline degree in regions where the melted polymer cools slower (deeper in the pool bed), which is directly associated with increased hardness values along the trough-thickness distance. Therefore, since the regions closer to the surface cool faster, the macromolecules have less time to organize, and crystals grow less, leading to a lower hardness in those regions compared to regions that cool slower. A slower cooling rate reduces the presence of amorphous regions, which are typically softer and more ductile. This justifies the hardness gradient according to the gradient of cooling rates. This structural dependence is particularly evident in samples processed at lower power and higher scanning speeds, where thermal input is better controlled, limiting polymer thermal degradation while enabling a uniform hardness gradient along the thickness. These findings are consistent with other laser-processed thermoplastic systems, where increased microhardness correlates with improved crystallinity and lower free volume in the HAZ [7,14,17,46].

## 4. Conclusions

This study evaluated the influence of laser parameters, specifically laser power and scanning speed, on the through-thickness microhardness of polypropylene plates. The results demonstrated that higher scanning speeds significantly improved the uniformity of hardness distribution. In contrast, higher power levels (300 W) produced a more pronounced heat-affected zone (HAZ), leading to deeper thermal penetration and structural changes in the material.

Consistent with previous studies using different laser systems, our findings confirm that high laser power combined with low scanning speed results in broader and deeper weld zones due to the accumulation of thermal energy and elevated internal temperatures within the polymer. In contrast, lower power and higher scanning speed favor the formation of narrower weld beads with more uniform hardness profiles and minimal thermal degradation, thus producing high-quality weld joints.

These findings reinforce the critical role of process parameter optimization in laser welding of thermoplastics, particularly when mechanical integrity and repeatability are required. Future research should investigate additional laser parameters, such as beam modulation, frequency, and focal position, and expand the study to other thermoplastic materials. Moreover, exploring the mechanical performance of the welded joints under variable environmental conditions and validating the results in industrial-scale trials would further support the implementation of this technique in real-world manufacturing contexts. Furthermore, this study could be expanded with other characterization methods, such as gel permeation chromatography (GPC) or size exclusion chromatography (SEC) to evaluate molecular weight variation due to chemical degradation, or differential scanning calorimetry (DSC) and X-ray diffraction (XRD) to analyze the crystalline degree changes on samples collected from different regions along the thickness plate. These other results can lead to more complex models that combine the prediction of temperature field, stress field, and melt flow field during heating and cooling with models that relate these fields to chemical thermal degradation and recrystallization. Hence, the results presented in this work may help validate these new models’ results.

## Figures and Tables

**Table 1:** Properties of the polypropylene (PP) adopted to this work.

Properties	Values
Density	0.92 g/cm^3^
Flammability	HB UL94
Young’s Modulus	1380 MPa
Yield Strength	30 MPa
Tensile Strength	33 MPa
Hardness (Shore D)	73 Scale D
Coefficient of Thermal Expansion (CTE)	$160\times 10^{-6}\;K^{-1}$
Thermal Conductivity	$0.22\;{Wm}^{-1}K^{-1}$
Continuous Service Temperature (CST)	0–100 °C
Melting Temperature	162–167 °C

**Table 2:** Laser welding robot parameters.

Printing Parameters	Values
Wave length λ	1030 ± 10 nm
Focal Distance	10 mm
Collimation Distance	150 mm
Beam Diameter	0.4 mm
Laser Power	200, 300 W
Speed	5, 10, 20, 30, 40, 50 mm/s
Focal Length	0 mm

**Table 3:** Average hardness and standard deviation across the thickness for different scanning speeds at 200 W laser power.

50 [mm/s]			40 [mm/s]			30 [mm/s]			20 [mm/s]			10 [mm/s]			5 [mm/s]		
Dist.	HV	HV	Dist.	HV	HV	Dist.	HV	HV	Dist.	HV	HV	Dist.	HV	HV	Dist.	HV	HV
[mm]	avg	std	[mm]	avg	std	[mm]	avg	std	[mm]	avg	std	[mm]	avg	std	[mm]	avg	std
0.158	0.574	0.103	0.154	0.597	0.119	0.158	0.571	0.091	0.820	0.667	0.144	0.357	0.541	0.216	0.559	7.517	0.105
0.322	0.570	0.062	0.292	0.575	0.084	0.266	0.596	0.120	1.070	0.636	0.094	0.607	0.680	0.179	0.809	8.503	0.156
0.572	0.616	0.078	0.542	0.609	0.061	0.516	0.644	0.137	1.320	0.650	0.116	0.857	0.619	0.171	1.059	9.497	0.145
0.822	0.641	0.068	0.792	0.643	0.075	0.766	0.672	0.157	1.570	0.633	0.074	1.107	0.646	0.178	1.309	9.197	0.124
1.072	0.650	0.055	1.042	0.657	0.088	1.016	0.674	0.118	1.820	0.649	0.090	1.357	0.679	0.134	1.559	10.030	0.187
1.322	0.645	0.069	1.292	0.650	0.092	1.266	0.636	0.074	2.070	0.669	0.102	1.607	0.712	0.169	1.809	10.020	0.208
1.572	0.638	0.031	1.542	0.676	0.105	1.516	0.636	0.096	2.320	0.660	0.112	1.857	0.664	0.085	2.059	10.883	0.193
1.822	0.645	0.027	1.792	0.711	0.118	1.766	0.681	0.099	2.570	0.685	0.084	2.107	0.700	0.109	2.309	10.787	0.168
2.072	0.662	0.068	2.042	0.692	0.083	2.016	0.684	0.115	2.820	0.686	0.108	2.357	0.724	0.172	2.559	10.743	0.195
2.322	0.680	0.061	2.292	0.724	0.087	2.266	0.709	0.118	3.070	0.684	0.085	2.607	0.751	0.211	2.809	9.997	0.110
2.572	0.699	0.086	2.542	0.656	0.068	2.516	0.703	0.105	3.320	0.692	0.098	2.857	0.682	0.092	3.059	10.180	0.118
2.822	0.720	0.087	2.792	0.677	0.052	2.766	0.707	0.109	3.570	0.701	0.108	3.107	0.696	0.089	3.309	10.413	0.136
3.072	0.719	0.091	3.042	0.716	0.091	3.016	0.666	0.113	3.820	0.674	0.109	3.357	0.739	0.117	3.559	10.630	0.156
3.322	0.697	0.028	3.292	0.719	0.100	3.266	0.714	0.139	4.070	0.698	0.077	3.607	0.745	0.115	3.809	10.360	0.110
3.572	0.712	0.085	3.542	0.708	0.096	3.516	0.695	0.081	4.320	0.718	0.101	3.857	0.742	0.119	4.059	10.117	0.138
3.822	0.710	0.107	3.792	0.707	0.094	3.766	0.704	0.074	4.570	0.734	0.107	4.107	0.727	0.118	4.309	10.587	0.155
4.072	0.719	0.112	4.042	0.709	0.121	4.016	0.687	0.045	4.820	0.707	0.113	4.357	0.711	0.079	4.559	10.533	0.140
4.322	0.711	0.102	4.292	0.707	0.090	4.266	0.718	0.108	5.070	0.697	0.082	4.607	0.774	0.104	4.809	10.623	0.139
4.572	0.709	0.108	4.542	0.712	0.110	4.516	0.722	0.106	5.320	0.759	0.197	4.857	0.729	0.125	5.059	10.557	0.112
4.822	0.696	0.070	4.792	0.717	0.119	4.766	0.745	0.120	5.570	0.743	0.113	5.107	0.736	0.119	5.309	10.613	0.122
5.072	0.717	0.109	5.042	0.706	0.068	5.016	0.711	0.067	5.820	0.754	0.166	5.357	0.746	0.141	5.559	10.093	0.058
5.322	0.733	0.099	5.292	0.706	0.094	5.266	0.711	0.104	6.070	0.773	0.185	5.607	0.766	0.109	5.809	10.620	0.123
5.572	0.705	0.099	5.542	0.688	0.069	5.516	0.709	0.105				5.857	0.762	0.173	6.059	10.483	0.135
5.822	0.702	0.093	5.792	0.708	0.080	5.766	0.727	0.130				6.107	0.752	0.153	6.309	10.003	0.112

**Table 4:** Average hardness and standard deviation across the thickness for different scanning speeds at 300 W laser power.

50 [mm/s]			40 [mm/s]			30 [mm/s]			20 [mm/s]			10 [mm/s]			5 [mm/s]		
Dist.	HV	HV	Dist.	HV	HV	Dist.	HV	HV	Dist.	HV	HV	Dist.	HV	HV	Dist.	HV	HV
[mm]	avg	std	[mm]	avg	std	[mm]	avg	std	[mm]	avg	std	[mm]	avg	std	[mm]	avg	std
0.170	0.572	0.019	0.216	0.595	0.028	0.167	0.560	0.069	0.123	0.646	0.093	0.441	0.521	0.054	0.434	0.584	0.040
0.420	0.590	0.075	0.466	0.573	0.044	0.577	0.554	0.084	0.533	0.574	0.065	0.691	0.613	0.068	0.844	0.654	0.047
0.670	0.612	0.098	0.716	0.619	0.064	0.987	0.563	0.070	0.943	0.607	0.045	0.941	0.583	0.069	1.254	0.591	0.051
0.920	0.655	0.048	0.966	0.621	0.064	1.397	0.630	0.059	1.353	0.644	0.070	1.191	0.636	0.087	1.664	0.590	0.093
1.170	0.644	0.058	1.216	0.639	0.045	1.807	0.640	0.069	1.763	0.655	0.065	1.441	0.683	0.100	2.074	0.637	0.076
1.420	0.639	0.055	1.466	0.639	0.052	2.217	0.624	0.051	2.173	0.632	0.044	1.691	0.650	0.041	2.484	0.650	0.055
1.670	0.650	0.075	1.716	0.650	0.061	2.627	0.622	0.060	2.583	0.634	0.043	1.941	0.658	0.062	2.894	0.665	0.059
1.920	0.673	0.062	1.966	0.659	0.050	3.037	0.639	0.074	2.993	0.657	0.056	2.191	0.695	0.051	3.304	0.665	0.068
2.170	0.681	0.063	2.216	0.690	0.026	3.447	0.660	0.068	3.403	0.661	0.056	2.441	0.655	0.074	3.714	0.654	0.048
2.420	0.681	0.100	2.466	0.705	0.022	3.857	0.661	0.080	3.813	0.661	0.049	2.691	0.712	0.071	4.124	0.657	0.075
2.670	0.669	0.033	2.716	0.662	0.044	4.267	0.657	0.063	4.223	0.658	0.039	2.941	0.674	0.023	4.534	0.634	0.071
2.920	0.695	0.049	2.966	0.677	0.089	4.677	0.657	0.056	4.633	0.666	0.046	3.191	0.676	0.053	4.944	0.662	0.035
3.170	0.682	0.058	3.216	0.701	0.043	5.087	0.645	0.076	5.043	0.673	0.038	3.441	0.714	0.050	5.354	0.656	0.063
3.420	0.698	0.065	3.466	0.680	0.049	5.497	0.642	0.078	5.453	0.685	0.050	3.691	0.704	0.074	5.764	0.661	0.077
3.670	0.696	0.047	3.716	0.692	0.055	5.907	0.667	0.098	5.863	0.685	0.051	3.941	0.691	0.060	6.174	0.683	0.051
3.920	0.708	0.044	3.966	0.697	0.032	6.317	0.637	0.078	6.273	0.680	0.059	4.191	0.650	0.062			
4.170	0.704	0.073	4.216	0.695	0.049	6.727	0.639	0.080	6.683	0.684	0.034	4.441	0.655	0.071			
4.420	0.707	0.085	4.466	0.676	0.065	7.137	0.620	0.093	7.093	0.663	0.069	4.691	0.653	0.056			
4.670	0.716	0.068	4.716	0.692	0.047	7.547	0.660	0.069	7.503	0.664	0.064	4.941	0.653	0.046			
4.920	0.732	0.089	4.966	0.695	0.058	7.957	0.648	0.087				5.191	0.688	0.046			
5.170	0.685	0.084	5.216	0.689	0.038	8.367	0.617	0.097				5.441	0.640	0.072			
5.420	0.687	0.092	5.466	0.705	0.048	8.777	0.617	0.110				5.691	0.643	0.056			
5.670	0.693	0.092	5.716	0.696	0.037	9.187	0.607	0.113				5.941	0.638	0.064			
5.920	0.682	0.086	5.966	0.697	0.046							6.191	0.645	0.056			
6.170	0.689	0.104	6.216	0.701	0.056							6.441	0.650	0.058			

## Data Availability

The original contributions presented in this study are included in the article. Further inquiries can be directed to the corresponding author.
